# Efficacy and tolerability of high dose "ethinylestradiol" in post-menopausal advanced breast cancer patients heavily pre-treated with endocrine agents

**DOI:** 10.1186/1477-7819-4-44

**Published:** 2006-07-11

**Authors:** Amit Agrawal, John FR Robertson, KL Cheung

**Affiliations:** 1 Professorial Unit of Surgery, City Hospital, University of Nottingham, Nottingham, NG5 1PB, UK

## Abstract

**Background:**

High dose estrogens (HDEs) were frequently used as endocrine agents prior to the introduction of tamoxifen which carries fewer side effects. Due to the development of resistance to available endocrine agents in almost all women with metastatic breast cancer, interest has renewed in the use of HDEs as yet another endocrine option that may have activity. We report our experience with one of the HDEs ("ethinylestradiol" 1 mg daily) in advanced breast cancer (locally advanced and metastatic) in post-menopausal women who had progressed on multiple endocrine agents.

**Patients and methods:**

According to a database of advanced breast cancer patients seen in our Unit since 1998, those who had complete set of information and fulfilled the following criteria were studied: (1) patients in whom further endocrine therapy was deemed appropriate i.e., patients who have had clinical benefit with previous endocrine agents or were not fit or unwilling to receive chemotherapy in the presence of potentially life-threatening visceral metastases; (2) disease was assessable by UICC criteria; (3) were treated with "ethinylestradiol" until they were withdrawn from treatment due to adverse events or disease progression.

**Results:**

Twelve patients with a median age of 75.1 years (49.1 – 85 years) were identified. Majority (N = 8) had bony disease. They had ethinylestradiol as 3^rd ^to 7^th ^line endocrine therapy. One patient (8%) came off treatment early due to hepato-renal syndrome. Clinical benefit (objective response or durable stable disease for ≥ 6 months) was seen in 4 patients (33.3%) with a median duration of response of 10+ (7–36) months. The time to treatment failure was 4 (0.5–36) months.

**Conclusion:**

Yet unreported, high dose "ethinylestradiol" is another viable therapeutic strategy in heavily pre-treated patients when further endocrine therapy is deemed appropriate. Although it tends to carry more side effects, they may not be comparable to those of other HDEs (such as diethylstilbestrol) or chemotherapy.

## Background

High dose estrogens (HDEs) were the alternative modality of endocrine therapy in advanced breast cancer besides surgical ablation of ovaries before the advent of modern endocrine agents. The discovery of tamoxifen relegated use of HDEs as first line endocrine therapy to history.

In almost all women with advanced breast cancer, duration of activity is shortened by development of resistance to endocrine agents. Interest, therefore, has renewed in the usage of HDEs towards the end of sequencing therapy after resistance to multiple endocrine agents. Usage of diethylstilboestrol (DES) in these patients heavily pre-treated with endocrine agents was reported recently by Lonning *et al *[1]. Yet unreported in literature [2], we demonstrate from our dataset that reasonable clinical efficacy and better tolerability can be obtained with high dose "ethinylestradiol" in these heavily pre-treated patients.

## Patients and methods

### Patients

Case notes of patients with advanced breast cancer (metastatic and locally advanced) since 1998 fulfilling the following criteria were reviewed:

• Estrogen receptor (ER) positive disease

• With disease assessable by UICC criteria [3]

• Patients have had previous endocrine therapies

• In whom further endocrine therapy was deemed appropriate i.e., those patients who have had clinical benefit with previous endocrine agents and were not fit or unwilling to receive chemotherapy in the presence of potentially life-threatening visceral metastases.

### Methods

Patients were treated with Ethinylestradiol (1 mg daily) until they were withdrawn from treatment either due to adverse events or disease progression.

Assessment of therapeutic response was made as per UICC criteria. Assessable lesions were deemed to have shown clinical benefit (CB) when they either had objective response in the form of complete response (CR) or partial response (PR); or had stable disease (SD) for ≥ 6 months [4,5]. Time to treatment failure (TTF) is the duration of treatment in months of patients who have failed on treatment including those with adverse events (not including patient still on treatment)

Time to progression (TTP) is the duration of treatment in months of patients who have progressed (not including patient still on treatment).

Duration of Response (DOR) is the duration of treatment in months of patients who have had CB.

## Results

Twelve patients with a median age of 75.1 years (49.1 – 85.0 years) were identified as shown in Table 1. Out of all these post-menopausal women, 3 patients had locally advanced breast cancer while the remaining 9 had metastatic breast cancer. Eight of the 9 patients with metastatic breast cancer had bony disease with bone only disease in 4 of them.

**Table 1:** Patient characteristics and tumor response to ethinylestradiol

**Patient Number**	**Age at Rx (years)**	**Sites of disease**	**Previous endocrine therapies**	**Response at 6 months**	**Duration of Treatment (months)**
1	85	Local	T, M, A, E, T	**PR**	7+
2	57	Local	T+G, M, Agt, Fr+G, T, A	**PR**	8
3	84	Bone, liver, mediastinal nodes	T, M, A, E	**PR**	12
4	76	Local	T, A, M	**SD**	36
5	80	Local, bone, pleura, ascites	T, F, A, M, E	PD	3
6	49	Bone	T+G, A+G, M, E+G	PD	6
7	79	Local, bone, lung	T, M, Agt, MPA, Fr	PD	2
8	74	Bone	M, E, T	PD	3
9	74	Bone	T, A, M, E	PD	2
10	84	Lung, pleura	A, M, E, F	PD	4
11	64	Bone	E, Lt, T	PD	6
12	64	Bone, liver	A, M	N/A*	0.5

Rx = Treatment; T = Tamoxifen; A = Anastrozole; E = Exemestane; M = Megestrol acetate; Lt = Letrozole; F = Fulvestrant; Agt = Aminoglutethimide; Fr = Formestane; MPA = Medroxyprogesterone acetate; G = Goserelin; N/A* = Not applicable as the patient was withdrawn due to side-effects.

The patients received ethinylestradiol as 3^rd ^to 7^th ^line endocrine therapy for advanced breast cancer. Four (no.1, 2, 3, 4) out of 12 patients had CB (33.3%) with a median DOR of 10 + (7 to 36) months. Objective response in the form of PR was seen in 3 (25%) patients (no. 1, 2, 3) while one patient (no. 4) had SD for 36 months.

The median TTP of the patients was 5 (2 to 36) months. One patient (no. 1) is still on treatment at 7+ months. Another patient (no. 12) came off treatment early due to hepato-renal syndrome. Therefore, the median TTF is 4 (0.5 to 36) months. Efficacy of ethinylestradiol is demonstrated in Table 1.

**Figure 1:**
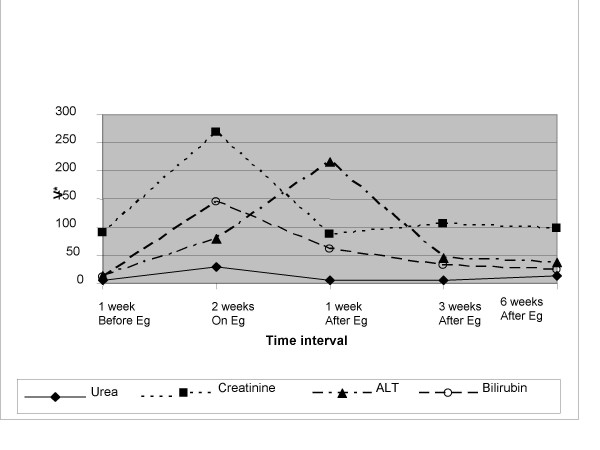
Changes in clinical chemistry on ethinylestradiol in patient (no. 12) with hepato-renal syndrome. ALT (Alanine amino-transferase); Bilirubin = Total Bilirubin; Eg = Ethinylestradiol; Before Eg = before commencement of Eg; After Eg = after withdrawal of Eg V* = Reference Ranges for values: Urea = 1.0–6.5 mmol/L; Creatinine = 60–120 μmol/L; ALT = 5–40 U/L; Total Bilirubin = 0–17 μmol/L.

Apart from the patient (no 12) who came off treatment in 2 weeks due to hepato-renal syndrome, none of the other patients had grossly deranged Liver Function Tests (LFTs) secondary to ethinylestradiol or any other reported side-effects. The deranged LFTs in this patient improved as soon as ethinylestradiol was stopped as shown in Figure 1.

## Discussion

### Efficacy

The first clinical trial of tamoxifen in post-menopausal women with advanced breast cancer was reported in 1971 by Cole *et al *[6]. They reported similar efficacy of tamoxifen compared with efficacy of DES or androgen in another trial. In a randomized clinical trial comparing nafoxidine (an erstwhile non-steroidal antagonist) and ethinylestradiol back in 1975, Heuson *et al*[7] demonstrated objective remission in 14% of 49 women on ethinylestradiol compared with 31% with nafoxidine. Massidda *et al*[8] reported objective remission in 50 % of patients treated with estrogens.

Beex *et al*[9] reported objective remission in 31% of ethinylestradiol treated patients compared with 33% of tamoxifen treated patients in a randomized study of 63 post-menopausal women with advanced breast cancer. The median duration of response and median survival times of ethinylestradiol and tamoxifen were similar: 12 months versus 11 months and 31 months versus 25 months respectively.

A randomized clinical trial of 143 women by Ingle *et al*[10] compared tamoxifen with DES in post-menopausal patients to determine their relative efficacy and toxicity. 99 women had received no prior systemic therapy and 44 had received previous chemotherapy. The objective response rate was higher in patients who received DES (41%) than in those who received tamoxifen (33%), though the difference was not significant (P = 0.37). In patients who had had no prior systemic therapy, the response rates with DES and tamoxifen were 44% and 38%, respectively (P = 0.55), and in those who had had previous chemotherapy, 32% vs. 23% respectively (P = 0.50). In addition, there was no significant difference between the two treatment groups on analysis of the time to treatment failure (medians: DES, 142 days; tamoxifen, 171 days). A recent update of this trial [11] reported significant (P = 0.039) survival advantage of patients on DES over tamoxifen (median survival of 3.0 years versus 2.4 years).

Therefore, most of the studies had proved beyond doubt similar efficacy of HDEs, if not greater when compared with early anti-estrogens including tamoxifen, there being no statistically significant difference. As because tamoxifen had a favourable side-effect profile, it was the preferred agent to estrogens [12,13].

An assorted collection of endocrine agents such as aromatase inhibitors, progestogens, pure anti-estrogens have since been in vogue. The development of resistance to all these agents though has proved to be the Achilles' heel of the whole gamut of endocrine therapy. Even if one agent is used after another as is the practice in sequencing therapy, each agent yields to the development of resistance. Therefore, the need for further endocrine therapy has rekindled interest in the use of HDEs as yet another endocrine option with possible activity.

The interest in the usage of HDEs after heavy pre-treatment with anti-estrogens comes from several in-vitro and animal studies. Masamura *et al *[14] showed in MCF-7 cells that long-term estrogen deprivation (akin to usage of anti-estrogens in humans) caused cells to develop estrogen hypersensitivity. Though cells replicated at a concentration of 10^-15 ^to 10^-14 ^M/L of estradiol, at a concentration of 10^-10 ^M/L, replication was inhibited. Osipo et a l [15] demonstrated apoptosis in long-term estrogen deprived, aromatase resistant breast cancer cell model (MCF-7:5C) at a concentration of 10^-11 ^M/L or more of estradiol. Similarly, in tamoxifen treated athymic mice serially transplanted with MCF-7TAM and MT2 tumor lines, Yao *et al *[16] showed that tumor regressed in response to estradiol. Later, both lines regrew on estradiol but the lines were then responsive to tamoxifen again.

The CB rate of 33% in our study is similar to published literature. However, our patients were not endocrine naïve patients as in previous studies. They have had at least 2 previous estrogen depriving therapies for advanced breast cancer and were at the end of the sequence of endocrine treatment lines. Lonning *et al*[1] recently demonstrated a high response rate to DES given at least after 2 (median of 4) previous estrogen depriving endocrine therapies. The authors demonstrated a CB rate of 38% in 32 patients and therefore, surmised that the estrogens administered in high doses represent a valuable alternative to chemotherapy in selected patients. Further data on HDEs may be available from a phase II study by WJ Gradishar at Robert Lurie Cancer Center (in collaboration with National Cancer Institute), Chicago, USA which commenced in August 2005 to determine clinical response and side-effects of high dose esterified estrogens (Menest™) in post-menopausal women with metastatic breast cancer that has failed previous hormone therapy.

### Tolerability

Side-effects are commoner with HDEs compared with anti-estrogens resulting in higher rate of withdrawal from therapy but they still appear to be more favorable than chemotherapeutic agents. Massidda *et al*[8] reported side-effects mainly as cholestatic liver abnormality. In the study by Beex *et al*[9], 2 patients were withdrawn because of hepatic impairment.

Lonning *et al*[1] reported a withdrawal rate of 18% (6 out of 32 patients) owing to side-effects. Similar toxicity rate was reported in patients receiving DES in study by Ingle *et al*[10]; 9 of 74 patients (12%) discontinued therapy solely because of adverse reactions.

One (= 8.3%) patient in our study was withdrawn due to the development of hepato-renal syndrome within 2 weeks of commencement of treatment. The derangement of hepatic function, however, reverted back to normality within 2 weeks of stopping treatment in spite of the fact that this patient had pre-existing liver metastasis. As the LFTs were normal in this patient before the commencement of therapy, early (within 2 weeks) and regular testing of LFTs may be imperative in patients treated with ethinylestradiol. The withdrawal rate of 8.3% is, however, less than 18% seen with DES [1] in similar heavily pre-treated patient group. There were no reported side-effects in the remaining patients.

Most of the side-effects attributable to estrogens are, however, due to the high clinical doses including the patient with hepato-renal syndrome in our study group. Osipo *et al*[15] and Jordan *et al*[17] have recently showed in their in-vivo study in athymic mice involving MCF-7 breast cancer cell lines that complete reversal of resistance to tamoxifen can be achieved with the use of low dose estrogen therapy. Based on their results, they suggest using an alternating treatment regimen, cycling antiestrogen with estrogen therapy to avoid drug-resistance.

In a clinical series by Pellegrini *et al *[18] in 1981, 19 patients unresponsive to conventional chemotherapy and chemo-hormone therapy were treated with an alternating sequential schedule of ethinylestradiol and medroxyprogesterone on the basis of correlations between hormones and estrogen and progestin receptors. Six patients had partial or complete remission and 5 others had minor responses.

## Conclusion

The resistance to endocrine therapy is evident in all lines of endocrine therapy with any of the available endocrine agents. Therefore, a virtual endpoint seems to appear wherein these patients are at the end of sequencing therapy with these endocrine agents. If these patients have had CB with endocrine therapy and if they have disease not suitable for chemotherapeutic agents or because the patients are not fit or unwilling; use of high dose ethinylestradiol may be beneficial. The side-effects are higher than existing endocrine agents but less when compared with chemotherapy. It perhaps may be useful to utilise high dose ethinylestradiol in the sequential endocrine therapy alternating with other endocrine agents although clinical efficacy of low dose estrogens in this context remains to be evaluated.

## Conflict of interest

The author(s) declare that they have no competing interests.

## Authors' contributions

**KLC **conceived this study. Patients were under care of **JFR **and **KLC**. **AA **collected data, performed analysis, drafted, revised and finalised the manuscript. **KLC **and **JFR **revised and approved of the contents of the manuscript. All authors read and approved the final manuscript.
